# Randomized study of remote telehealth genetic services versus usual care in oncology practices without genetic counselors

**DOI:** 10.1002/cam4.3968

**Published:** 2021-06-08

**Authors:** Cara N. Cacioppo, Brian L. Egleston, Dominique Fetzer, Colleen Burke Sands, Syeda A. Raza, Neeraja Reddy Malleda, Elisabeth McCarty Wood, India Rittenburg, Julianne Childs, David Cho, Martha Hosford, Tina Khair, Jamil Khatri, Lydia Komarnicky, Trina Poretta, Fahd Rahman, Satish Shah, Linda J. Patrick‐Miller, Susan M. Domchek, Angela R. Bradbury

**Affiliations:** ^1^ Penn Telegenetics Program University of Pennsylvania Philadelphia PA USA; ^2^ Fox Chase Cancer Center Biostatistics and Bioinformatics Facility Temple University Health System Philadelphia PA USA; ^3^ Department of Medicine Division of Hematology‐Oncology University of Pennsylvania Philadelphia Pennsylvania USA; ^4^ Shore Cancer Center Somers Point NJ USA; ^5^ Cape Regional Medical Center Cape May Court House NJ USA; ^6^ Union Hospital Elkton MD USA; ^7^ Gettysburg Cancer Center Gettysburg PA USA; ^8^ Drexel University Cancer Center Philadelphia PA USA; ^9^ Kennedy Cancer Center Sewell NJ USA; ^10^ Center for Clinical Cancer Genetics and Global Health The University of Chicago Chicago IL USA; ^11^ Abramson Cancer Center University of Pennsylvania Philadelphia PA USA; ^12^ Department of Medical Ethics and Health Policy University of Pennsylvania Philadelphia PA USA

**Keywords:** alternative service delivery, cancer genetics, cancer predisposition syndromes, genetics, genetic counseling, genetic testing, telegenetics, telehealth, telemedicine

## Abstract

**Purpose:**

To examine the benefit of telehealth over current delivery options in oncology practices without genetic counselors.

**Methods:**

Participants meeting cancer genetic testing guidelines were recruited to this multi‐center, randomized trial comparing uptake of genetic services with remote services (telephone or videoconference) to usual care in six predominantly community practices without genetic counselors. The primary outcome was the composite uptake of genetic counseling or testing. Secondary outcomes compare telephone versus videoconference services.

**Results:**

147 participants enrolled and 119 were randomized. Eighty percent of participants in the telehealth arm had genetic services as compared to 16% in the usual care arm (OR 30.52, *p* < 0.001). Five genetic mutation carriers (6.7%) were identified in the telehealth arm, compared to none in the usual care arm. In secondary analyses, factors associated with uptake were lower anxiety (6.77 vs. 8.07, *p* = 0.04) and lower depression (3.38 vs. 5.06, *p* = 0.04) among those who had genetic services. There were no significant differences in change in cognitive or affective outcomes immediately post‐counseling and at 6 and 12 months between telephone and videoconference arms.

**Conclusion:**

Telehealth increases uptake of genetic counseling and testing at oncology practices without genetic counselors and could significantly improve identification of genetic carriers and cancer prevention outcomes.

## Graphical abstract

Providing remote telehealth genetic services increases uptake of genetic counseling and testing at oncology practices without genetic counselors. These data highlight the value of telehealth strategies to significantly improve identification of genetic carriers and cancer prevention outcomes.

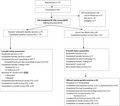

## 1 INTRODUCTION

Genetic testing for cancer predisposition has become standard practice,^1^ yet many patients do not have access to genetic services.^2^ Currently, genetic services are geographically limited, requiring many patients to travel long distances to referral centers.^2^ Telehealth can improve access,^3^, ^4^ provide cost and time savings^5^, ^6^ increase convenience, and provide a safe option in times of public health risks (e.g. pandemics).

There are several randomized studies that have compared uptake of testing and patient‐reported outcomes with telephone services as compared to in‐person services. These studies reported phone is no worse than in‐person counseling for several patient‐reported outcomes (e.g., knowledge, distress), although uptake of testing was lower in the phone arms and some suggest that there are remaining gaps in access and further research is needed.^7^, ^8^, ^9^, ^10^ Studies comparing fully remote real‐time video conferencing counseling to in‐person are more limited. The existing published studies are heterogeneous in setting and delivery, most are not randomized, have a small sample size and/or have limited patient‐reported outcomes.^4^, ^5^ Furthermore, there are no studies comparing remote telehealth genetic services (i.e., telephone or videoconferencing) to usual care options in oncology practices without genetic counselors. While some studies have utilized in‐person services as the non‐randomized comparison arm,^5^, ^7^, ^8^, ^9^, ^11^ we propose that the appropriate comparison is usual care, which in these communities includes patients traveling to a regional expertise center or receiving testing with local non‐genetics providers. In this multicenter randomized study, we sought to evaluate if providing remote telehealth services can increase uptake of genetic services compared to usual care. Additionally, we sought to evaluate patient outcomes of phone as compared to videoconference services in predominantly community practices.

## 2 METHODS

### 2.1 Study design

This is a three‐arm randomized study of remote services (phone or videoconference) compared to usual care. Our primary hypothesis was that patients in the remote services arm would have significantly higher uptake of pre‐test counseling and testing as compared to usual care. Our secondary hypotheses were that remote videoconference services would be associated with greater decreases in distress (state anxiety and cancer‐specific distress) and higher satisfaction (with genetic services) as compared to remote phone services.

### 2.2 Participants

The University of Pennsylvania (UPenn) Institutional Review Board approved the study. Informed written or verbal consent was obtained from all participants. Participants were recruited from August 2015 to December 2018 (NCT02517554).^12^ Sites included regional practices from different healthcare systems where genetic services were not available on site, including Kennedy Cancer Center (New Jersey), Union Hospital (Maryland), Drexel University Cancer Center (Pennsylvania), Shore Cancer Center (New Jersey), Gettysburg Cancer Center (Pennsylvania), and Cape Regional Medical Center (New Jersey).

Participants were identified by site research staff and included English‐speaking adults meeting current National Comprehensive Cancer Network^®^ criteria for cancer genetic testing. Site staff and providers were provided an eligibility checklist aligning with NCCN criteria and patients were identified in oncology clinics by research staff or providers consistent with where they had previously identified patients for genetic testing prior to the study. All participants were informed they met criteria for genetic testing based on their personal and family history. They were consented to the study by study site staff and then referred to the UPenn research team for the remainder of study procedures. The cost of testing was covered by insurance or self‐pay.

### 2.3 Randomization

After completing the baseline survey, participants were randomly assigned to one of two remote telehealth service arms (telephone or real‐time videoconference) or usual care, stratified by site, sex, and family using a permuted block design. Randomization was adjusted from 1:1:1 to 1:1:2 to achieve adequate enrolment to meet our primary outcomes.

### 2.4 Procedures

UPenn research staff provided participants an informational flyer with contact information for usual care or telehealth genetic services according to their study arm (Figure S1). In all arms, participants needed to take the initial step to contact the programs.

#### 2.4.1 Usual care arm

Participants in the usual care arm were provided an informational flyer listing contact information for several options for genetic services in their area, reflective of testing options that existed prior to the start of the study. These options were similar among sites, although the specific local referral programs varied. This included: (a) the option to drive to the Penn Cancer Risk Evaluation Program (the face‐to‐face clinical program at UPenn) and any other local genetic programs the site had used prior to study start. Other options included: (b) the National Society of Genetic Counselors, which provides a list of genetic providers by zip code, (c) 1‐800‐4‐CANCER, an information line provided by the National Cancer Institute, which can provide information on local services, and d) that patients could also inquire with their current health care providers (Figure S1a). Participants were contacted by research staff to confirm that they received the flyer and understood the information.

Participants in the usual care arm were contacted 6 months after randomization to assess if they had genetic counseling and testing (see 6‐Month Status Survey below). Those who had not received genetic testing were provided the option for remote telehealth services, in a wait‐list design and randomized 1:1 to phone or videoconference. They were not informed at their initial randomization that this would be available to them.

#### 2.4.2 Remote telehealth services arms (phone and real‐time videoconference)

Participants in the remote services arm were similarly provided an information flyer describing how to contact Penn Telegenetics to schedule remote services (Figure S1b,c). Again, all participants were contacted to confirm that they received the information sheet, but in both arms participants had to take the step to contact Penn Telegenetics. Appointments were scheduled at their oncology site to meet with a genetic counselor by videoconference or telephone. The private room included a telephone with speaker capabilities and a computer with links to HIPAA compliant videoconferencing software (MediSprout, Vidyo, and/or BlueJeans).

Remote telehealth services were delivered by three genetic counselors licensed per state guidelines. Genetic counseling services were covered by research funds and not submitted for insurance billing. Standardized communication protocols, visual aids and counseling checklists were utilized.^7^, ^13^ Mean fidelity to checklists was 96.4% for pre‐test and 96.3% for disclosure sessions. All genetic testing was consistent with standard‐of‐care clinical testing and billed to insurance. Participants returned to their site for result disclosure with the genetic counselor via their randomization arm, although participants in the telephone arm could receive results at home. Participants were recommended to follow‐up with their physician to discuss medical management recommendations.

### 2.5 Primary outcome measures

The primary protocol‐specified endpoint was a composite variable indicating whether a person had pre‐test counseling or genetic testing (defined as “genetic services”), to account for patient declining testing based on informed choice. Uptake of services were obtained through study records for the remote telehealth services arms and through a telephone administered 6‐Month Status Survey for the usual care group. The Status Survey queried completion of each outcome, date, and provider, and explored barriers and reasons if services were not completed.

### 2.6 Secondary outcome measures

Participants completed a baseline survey (T0) prior to randomization. This included the patient‐reported outcomes below as well as assessment of health literacy,^14^ and health behaviors.^15^, ^16^, ^17^ Participants in the remote telehealth service arms completed questionnaires 3–7 days after their pre‐test counseling (T1) and disclosure (T2), and at 6 (T3), and 12 months (T4). Surveys were self‐administered by REDCap software, paper, or phone. Our studies evaluating delivery innovation in genetic services have been informed by our conceptual model grounded in the Self‐regulation Theory of Health Behavior,^18^ including potential risks of telehealth communication, (e.g. poorer understanding of results, greater short‐term distress, and poorer behavioral outcomes^7^, ^19^).

*Knowledge of genetic disease* (T0‐T4) was evaluated using an 18‐item scale adapted from the ClinSeq knowledge,^20^, ^21^ (Cronbach's *α* = 0.78–0.92).

*Cancer*‐*specific distress* (T0‐T4) was evaluated with 14 items of Impact of Events Scale (IES) evaluating frequency of thoughts and feeling about cancer.^22^ We excluded one item lacking face validity in our population (“I felt as though it was not real”), (Cronbach's *α* = 0.88–0.91).

*General anxiety and depression* (T0‐T4) were assessed with the 14‐item Hospital Anxiety and Depression Scale (HADS),^23^ (Cronbach's *α* = 0.88–0.93).

*Multidimensional responses to genetic testing*, including positive responses (Cronbach's *α* = 0.63–0.79), negative responses (6‐items, Cronbach's *α* = 0.85–0.89), and uncertainty (9‐items, Cronbach's *α* = 0.64–0.80) were assessed with the Multi‐dimensional Impact of Cancer Risk Assessment Questionnaire (MICRA).^24^

*Satisfaction with genetic services* (T1–T2) was evaluated with a 12‐item scale evaluating participants’ cognitive and affective perceptions of their genetic counseling and testing experience,^13^ (Cronbach's *α* = 0.75–0.85).

*Satisfaction with telemedicine* (T3 and T4) was assessed with 10‐items adapted from Dick et al. and utilized in our prior research,^13^, ^25^ (Cronbach's *α* = 0.57–0.70).

### 2.7 Statistical analysis

The primary protocol‐specified endpoint was a composite variable indicating whether a person had pre‐test counseling or genetic testing. The primary comparison was the usual care arm versus the combined telehealth arm (telephone and video conference as one group). Target accrual was 70 patients in the usual care arm and 140 patients in the combined telehealth arms. This provided 91% power to detect a difference in testing of 40% (usual care arm) versus 65% (telehealth arms). This assumed a 5% Type I error rate (2‐sided) and the use of Fisher's Exact test.

The difference in uptake between the arms was much greater than expected by our power calculation, thereby meeting our primary objective using a Fisher's Exact Test. To be conservative, we hence defaulted to our secondary analytic approach of using logistic regressions that controlled for potential baseline confounders that were not balanced among arms, including literacy, baseline knowledge, baseline depression, previous history of cancer (yes/no), education (high school, some college, college), and income (<$50,000/year versus $50,000+). We accounted for missing data by using a multiple imputation approach with 100 imputed datasets.^26^

For other secondary analyses, we used linear or logistic regressions with the multiply imputed data. We controlled for remote versus usual care arm when comparing baseline variables between those who did and did not take up genetic services. One exception was the comparison of carriers identified by remote services versus usual care, in which we used a Fisher's exact test in the non‐imputed data due to the small number of carriers. In regressions of psychosocial responses, we controlled for the imbalanced variables as described above. The criteria for statistical significance was *p* < 0.05 and for marginal statistical significance was 0.05 < *p* < 0.10. We used the IVEWare macro in SAS 9.4 to analyze the data.

## 3 RESULTS

### 3.1 Study participants

**FIGURE 1:**
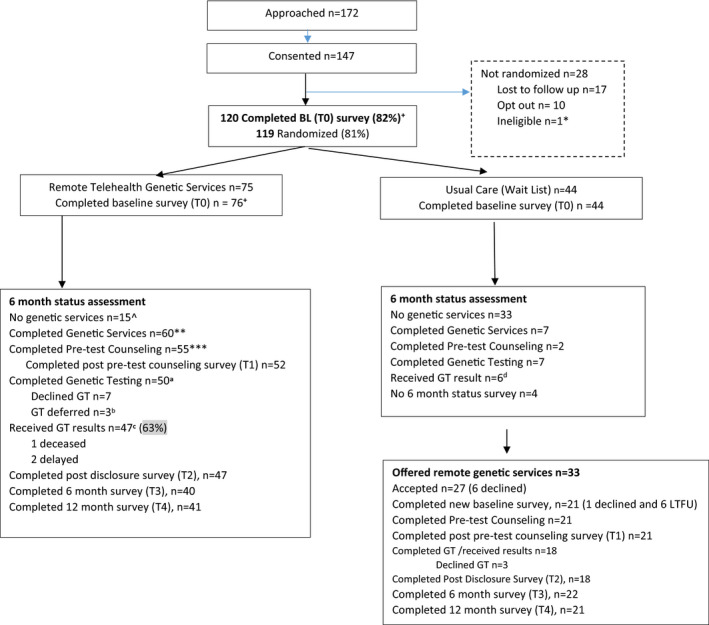
Study consort. *One enrolled participant was found to be ineligible and not randomized. ^+^One participant completed T0 survey but we were unable to reach the participant to complete randomization. ^++^Randomization was initially 1:1:1 (remote phone: remote videoconference: usual care), but was changed to 1:1:2 to achieve adequate enrollment to meet our primary outcomes. ^One participant deceased. **Includes five participants who had external testing but not through our remote services (e.g. not per protocol). ***Does not include five participants who had external testing, because we can't confirm outside pre‐test counseling. **^a^** Includes five participants who had external testing and 45 remote participants who had V1 and blood draw within 6 months of randomization. ^b^Testing deferred includes: (1) two participants who waited for relatives to test first and did not get genetic testing; one participant waited for mother's genetic testing but did finally have genetic testing through remote services. ^c^One participant died before results could be disclosed; two participants had disclosures at 7 and 8.8 months post randomization, respectively. ^d^One UC participant received results at 7.3 months

One hundred forty‐seven participants enrolled (85% of approached), 120 (82%) completed T0 and 119 were randomized (see Figure 1). There were no significant differences in baseline characteristics between those who declined and enrolled. Twenty‐seven participants were not randomized due to opting‐out or loss to follow‐up. Reasons for opting‐out included: not interested, not feeling at risk for hereditary cancer, time constraints, and concerns about cost. Participant characteristics are outlined in Table 1. Eighteen percent of patients were non‐white and 64% had less than a college degree. Despite randomization, those in the remote telehealth services arm were less likely to have had cancer, more likely to have college or more education and higher income and higher health literacy and knowledge at baseline. We adjusted for these baseline differences in analyses.

**TABLE 1:** Participant characteristics (*n* = 119)

Variable	Remote telehealth genetic services *n* = 75	Usual care *n* = 44
Age, mean, SD	52.4 (13.0)	55.0 (11.6)
Female gender no. (%)	70 (93.3)	40 (90.9)
Race no. (%)		
White	60 (80.0)	37 (84.1)
Black	9 (12.0)	6 (13.6)
Other	6 (8.0)	1 (2.3)
Education^a^		
College degree or higher	28 (37.3)	15 (34.1)
Some college/associate degree	33 (44.0)	11 (25.0)
Some/completed high school	14 (18.7)	18 (40.9)
Marital status		
Married/domestic partnership	46 (61.3)	25 (56.8)
Divorced/separated/widowed	17 (22.7)	14 (31.8)
Single	12 (16.0)	5 (11.4)
History of cancer		
Yes^b^ (%)	44 (58.7)	35 (79.5)
Breast	28 (63.6)	22 (62.9)
Colorectal	4 (9.1)	2 (5.7)
Ovary	2 (4.6)	0 (‐)
Multiple primaries	4 (9.1)	4 (11.4)
Other	6 (13.6)	7 (20.0)
Previous Limited Genetic Testing*	5 (6.7)	2 (4.5)
Income level^c^		
≥$50,000	47 (66.2)	16 (41.0)
<$50,000	24 (33.8)	23 (59.0)
Missing	4	5
Community site		
Cape regional medical center	3 (4.0)	2 (4.6)
Drexel college of medicine	2 (2.7)	1 (2.3)
Gettysburg cancer center	24 (32.0)	12 (27.3)
Kennedy health system	2 (2.7)	2 (4.6)
Shore cancer center	22 (29.3)	14 (31.8)
Union hospital	22 (29.3)	13 (29.6)

	Mean (SD)	Mean (SD)
Literacy score^d^, score range 0–12	10.1 (2.5)	11.6 (2.7)
General depression, score range: 0–21	3.7 (2.9)	4.8 (4.0)
General anxiety, score range: 0–21	7.2 (3.5)	7.6 (3.7)
Cancer‐specific distress	21.1 (13.8)	23.1 (16.4)
Score range: 0–70		
Knowledge of genetic disease^e^	50.4 (16.0)	40.6 (22.7)
Score range: 0–87		

^a^ *p* = 0.023.

^b^ *p* = 0.022.

^c^ *p* = 0.025.

^d^ *p* = 0.052.

^e^ *p* = 0.007.

* Previous limited genetic testing = BRCA 1/2 (5), PMS2 sequencing (1), panel of high risk genes (1).

### 3.2 Uptake of genetic services

At 6 months, 80% of participants in the remote telehealth arm had genetic services as compared to 16% in the usual care arm (OR 30.5, *p* < 0.001, Table 2). This included a higher likelihood of both counseling (OR 74.5, *p* < 0.001) and testing (OR 11.6, *p* < 0.001). Most (84%) patients had a multi‐gene panel, and this did not differ between arms. Five genetic mutation (6.7%) carriers were identified in the remote services arm (two *MUTYH*, two *BRCA2*, and one *ATM*), and none in the usual care arm.

**TABLE 2:** Uptake of genetic services at 6 months

	Remote services *n* = 75 *N* (%)	Usual care *n* = 44 *N* (%)	*p*
Uptake of genetic services	60 (80.0)	7 (15.9)	<0.001
Uptake of genetic counseling^a^	55 (73.3)	2 (4.5)	<0.001
Uptake of genetic testing^b^	50 (66.7)	7 (15.9)	<0.001
Genetic carriers^c^	5 (6.7)	0 (‐)	0.16

Note

Differences between arms controlled for baseline differences in baseline knowledge, literacy, depression, history of cancer, education and income.

^a^ Genetic counseling with a licensed genetic counselor.

^b^ Includes five patients in remote services arm who had usual care genetic testing (all negative results), not through remote services consistent with an intention‐to‐treat approach.

^c^ BRCA2 (2), ATM, MUTYH (2).

After the 6‐Month Status Survey, 21 usual care participants were offered remote telehealth services in a wait‐list design. Uptake of genetic services did not differ significantly among those randomized to videoconference as compared to phone, either before or after inclusion of the wait list arm (Table 3).

**TABLE 3:** Uptake of genetic services by phone versus videoconference remote services^a^

Participants initially randomized to remote services (*n*=75)			
	Phone (*n* = 37)	Videoconference (*n* = 38)	*p*
Uptake of genetic services	28 (75.7)	32 (84.2)	0.85
Pre‐test counseling	26 (70.3)	29 (76.3)	0.86
Completed genetic testing^b^	22 (59.5)	29 (76.3)	0.87
Declined genetic testing	4 (10.8)	3 (7.9)	
Ineligible for genetic testing^c^	2 (5.4)	0 (‐)	
Lost to follow‐up/withdrew	9 (19.1)	6 (15.8)	
Carriers identified	3 (8.1)	2 (5.3)	0.8

All participants randomized to remote services, including wait list participants (*n* = 96)			
	Phone (*n* = 47)	Videoconference (*n* = 49)	*p*
Uptake of genetic services	38 (80.9)	43 (87.8)	0.82
Pre‐test counseling	36 (76.6)	40 (81.6)	0.96
Completed genetic testing^b^	30 (63.8)	39 (79.6)	0.78
Declined genetic testing	6 (12.8)	4 (8.2)	
Ineligible for genetic testing^c^	2 (4.3)	0 (‐)	
Lost to follow‐up/withdrew	9 (19.1)	6 (12.2)	
Carriers identified	3 (6.4)	2 (4.1)	0.64

^a^ Includes those whose genetic testing and/or results were returned after 6 months (*N* = 3).

^b^ Includes participants who had testing (all negative results) through their physician (not through remote services) two in phone arm, three in VC arm.

^c^ Ineligible for genetic testing due to previously completed panel testing identified after enrollment.

### 3.3 Factors associated with uptake of genetic services

We evaluated baseline factors associated with uptake of genetic services among all participants (*n* = 119) and adjusted for study arm. Uptake before waitlist re‐randomization was associated with lower general anxiety (6.77 vs. 8.07, *p* = 0.04) and depression (3.38 vs. 5.06, *p* = 0.04) among those who had genetic services.

Among usual care participants who provided a reason for not having genetic services (*n* = 34), the most frequently reported barriers included not having enough guidance on the information sheet (23.5%) (e.g. referral numbers were not enough to activate behavior), no time or competing priorities (20.6%), cost/insurance concerns (17.6%), not interested or no perceived utility (11.8%), and travel distance (11.8%). Other reasons included not recalling receiving the information sheet, being told by a healthcare provider they do not need testing, scheduling or referral challenges, and physical disability.

### 3.4 Patient outcomes with telephone versus videoconference services

In secondary analyses comparing patient reported outcomes with genetic services provided by videoconference as compared to telephone, there were no significant differences in change in cognitive or affective outcomes both immediately post‐counseling and at 6 and 12 months (Tables S1 and S2).

## 4 DISCUSSION

Disparities in access to genetic services have been identified as a significant challenge,^2^ especially for individuals in rural areas and minority populations. This randomized trial provides evidence that offering remote phone or videoconference telehealth services in community‐based oncology clinics increases uptake of genetic services. Our study also identifies favorable patient‐reported outcomes of telehealth genetic services, suggesting viable delivery models to improve adoption of genetic testing guidelines.

To our knowledge, this is the first study to compare uptake of genetic services to usual care options in oncology practices without genetic counselors. Other studies have shown equal patient reported outcomes but lower uptake of remote telehealth (predominately phone) services when compared to in‐person genetic counseling.^8^, ^9^, ^27^, ^28^ One randomized study found a higher attendance for those receiving in‐person counseling provided by a traveling genetic counselor compared to those receiving counseling by videoconference.^5^ Yet, having counselors travel to community sites is costly, time‐consuming, and becoming less common. Thus, most practices do not have access to on‐site genetic services and usual care is the most relevant real‐world comparison. We found greater uptake of genetic services with remote telehealth delivery when compared to usual care options, more accurately illustrating the impact of offering remote options in practices without genetic services. In all arms, participants had to take the first step of contacting the program. Some in the usual care arm may not have felt comfortable making the first call to these programs, but this represents what would have been offered prior to the study. Perceptions about the ease of telehealth or comfort of receiving services in their local clinic, as opposed to going to an outside usual care clinic, may have been an additional benefit of telehealth services.

We identified several patient reported barriers to accessing usual care genetic services, including lack of relevance and utility, limited knowledge about genetic counseling, and concerns about cost and insurance coverage.^29^, ^30^, ^31^ Additionally, participants reported not having time, having competing priorities or physical disabilities, which highlight unique challenges that telehealth may address. Our data also suggests that patients with anxiety and depression may be less likely to consider genetic testing, which is consistent with some but not all studies.^32^, ^33^ More research is needed to better understand psychological predictors of genetic testing in diverse patient populations. While offering telehealth services can increase uptake, additional barriers to genetic counseling and testing remain.

Prior studies have reported high satisfaction and acceptance of telehealth services by participants,^11^, ^13^, ^34^ but most have reported limited cognitive and affective outcomes and did not include multi‐gene panel testing.^8^, ^11^, ^28^ This study included discussion of multigene panel testing and a wide range of cancer syndromes, consistent with current testing options. Overall, our patient reported outcomes are similar to other studies reporting favorable patient reported outcomes in the era of multi‐gene panel testing, including small increases in knowledge, no significant increases in distress, and small or no changes in patient reported uncertainty.^7^, ^15^, ^35^, ^36^, ^37^, ^38^

Equally important, we found no significant differences in multiple cognitive and affective outcomes between the telephone and videoconference arms. To date, there is only one small randomized study comparing telephone and video genetic counseling in veterans undergoing limited testing for polyposis reporting higher uptake of genetic counseling, increased convenience with telephone services, and similar knowledge and satisfaction outcomes between the two arms.^34^ In contrast, we found no difference in uptake between telephone and videoconference services. While there are few studies comparing telephone to videoconference services in clinical genetics, there are many studies comparing telehealth outcomes in other areas of medicine; although, outcomes evaluated vary widely across the studies.^39^, ^40^, ^41^

Our study provides clinicians with evidence of the positive impact remote telehealth services may offer their practice in terms of access, uptake of services, and patient outcomes. During the current pandemic, telehealth has been even more widely adopted and comfort among providers and patients is expected to increase. Thus, uptake of remote services could be even higher and patient reported outcomes even better than we report in this study. Additionally, data regarding the benefits of phone as compared to remote videoconference services will be helpful for establishing best practices. While remote phone services address disparities in access to adequate internet access, whether phone services will be reimbursed at similar rates to remote videoconference will be critical to long‐term implementation. Lack of parity in reimbursement could exacerbate already existing health care disparities.

We acknowledge several limitations of our study. Although a multicenter study of a representative population of patients from practices without genetic counselors, our overall sample size was relatively small. While this sample size was sufficiently powered for analyzing uptake outcomes, our study was not powered for secondary comparisons of phone to videoconferencing, and larger studies are needed to draw firm conclusions about any potential differences between these modalities. While those offered remote services at six months were not previously aware that this would be offered, providing this opportunity at six months (wait‐list design) may have introduced an additional “cue to action” in this group. Additionally, we accepted self‐report of genetic services in the usual care arm. We also had missing data in four participants in the usual care arm, although this is only 9% of participants in this arm and would not change the inferences and conclusions. Our population of patients treated in community practices was more diverse than many studies in cancer genetics, but still had smaller representation of non‐white participants. Additionally, the intervention does not address the challenges that many practices face in identifying eligible patients and we cannot confirm that there may have been some eligible patients not approached. This remains a challenge of implementing genetic testing in clinical care and combining with novel ways to identify all eligible patients could also improve uptake of genetic testing in community practices.

In conclusion, providing remote genetic services, by phone or videoconference, increases uptake of genetic counseling and testing in oncology patients without access to genetic counselors. These data highlight the value of telehealth strategies to significantly improve uptake of guidelines for genetic testing and support further expansion of telehealth strategies, particularly as public health events provide increasing indications for remote medical services.

## CONFLICT OF INTEREST

Dr. Angela Bradbury has served on advisory boards for AstraZeneca and Merck. There are no other conflicts of interest to disclose.

## AUTHOR CONTRIBUTIONS

Cara N. Cacioppo, Angela R. Bradbury, Dominique Fetzer, Colleen Burke Sands, and Brian L. Egleston contributed to writing the original draft and review and editing. All authors reviewed and approved the manuscript. Brian L. Egleston, Cara N. Cacioppo, Angela R. Bradbury, Colleen Burke Sands, Syeda A. Raza, and India Rittenburg contributed to data curation and formal analysis. Cara N. Cacioppo, Elisabeth McCarty Wood, and Neeraja Reddy Malleda contributed to methodology and investigation. Angela R. Bradbury, Susan M. Domchek, Colleen Burke Sands, and Linda J. Patrick‐Miller contributed to conceptualization and methodology. Colleen Burke Sands, Angela R. Bradbury, and Dominique Fetzer contributed to project administration and supervision. Susan M. Domchek and Angela R. Bradbury secured funding for this study. Julianne Childs, David Cho, Martha Hosford, Tina Khair, Jamil Khatri, Lydia Komarnicky, Trina Poretta, Fahd Rahman, and Satish Shah served as sub‐PIs on the study at participating clinical sites and contributed to investigation.

## Supporting information

Fig S1

Table S1

Table S2

## Data Availability

This was an institutionally funded study. Readers may request de‐identified data and a data dictionary from the corresponding author. A data use agreement should be signed by those who request the data. The data use agreement would limit unapproved uses.
