# Supplementary material for: Randomized study of remote telehealth genetic services versus usual care in oncology practices without genetic counselors
Source: Cancer Med. 2021 Jun 8;10(13):4532–41. doi: 10.1002/cam4.3968 (PMC8267134; doi:10.1002/cam4.3968)
Supplement: Supplementary file 1 — Fig S1 [file CAM4-10-4532-s001.docx]

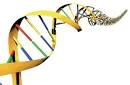

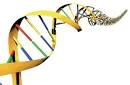
As part of the TeleGenetics Clinic Study, you have been randomly assigned to
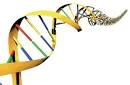
Usual Care. Next Step: you can use the sources below for information on how to receive genetic services in your area.

Your primary care provider: This could be your family doctor or nurse practitioner, or gynecologist if you are a woman.


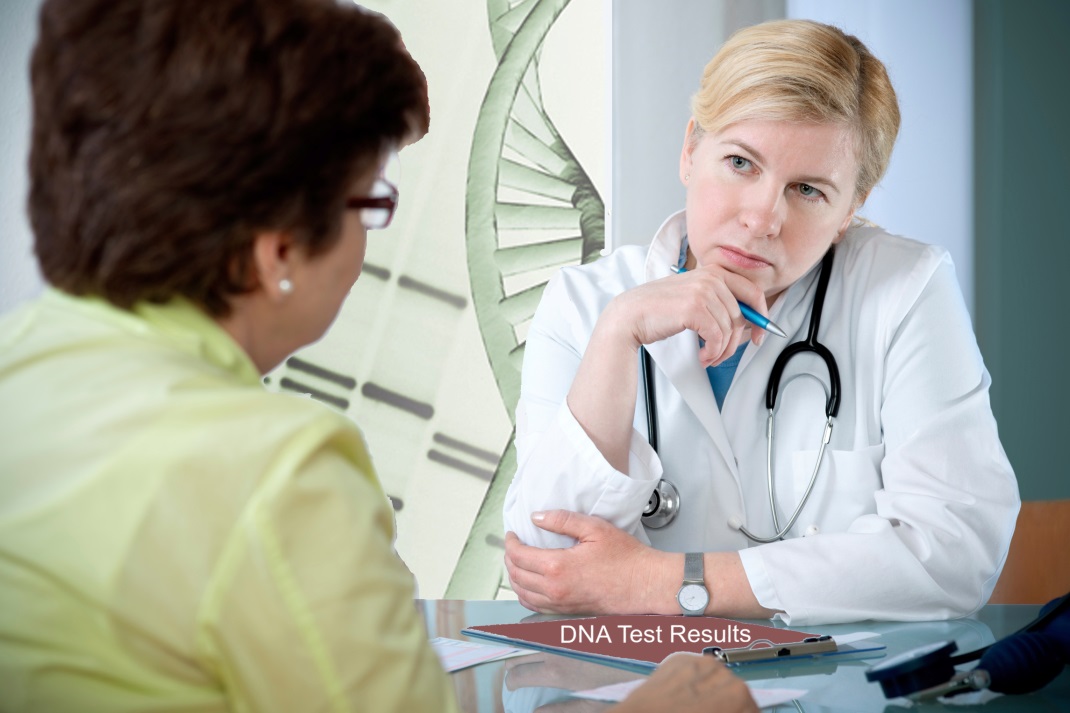


You can also find a genetic counselor in your area. Visit the National Society of Genetic Counselors website: [www.NSGC.org](http://www.NSGC.org)

Or call 1-800-4-CANCER

(1-800-422-6237)

You can call the Cancer Risk Evaluation Program at Abramson Cancer Center at the University of Pennsylvania.

215-349-9093

If you have any questions, contact:

[NAME OF SITE STAFF, PHONE NUMBER AND EMAIL]


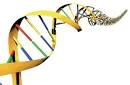
As part of the TeleGenetics Clinic Study, you have been randomly assigned to receive genetic services by Videoconference.

You will meet with a Penn Medicine genetic counselor by videoconference at the [NAME OF INSTITUTION]. Please contact the Penn TeleGenetics Clinic Study team to schedule your appointment:

[NAME OF RESEARCH TEAM, PHONE NUMBER AND EMAIL]


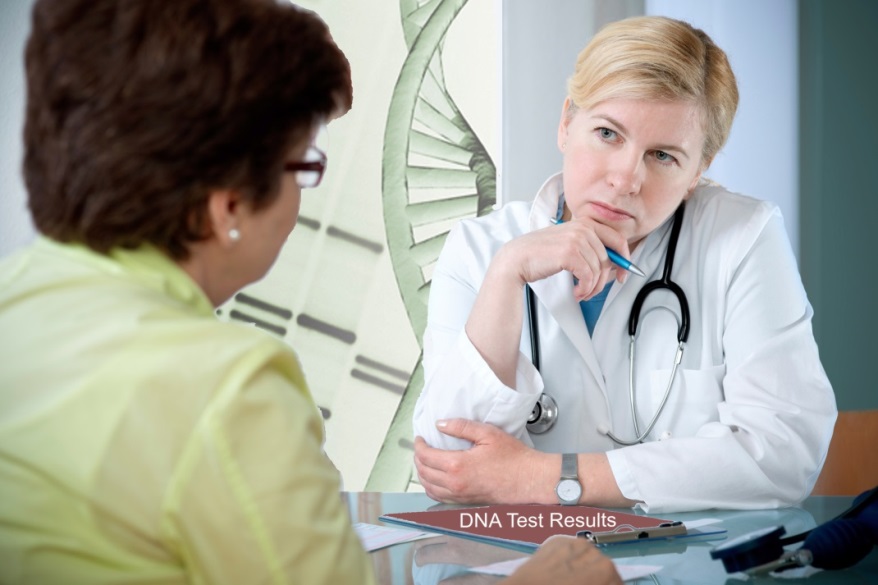


If you have any questions, contact:

[NAME OF SITE STAFF, PHONE NUMBER AND EMAIL]


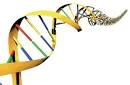
As part of the TeleGenetics Clinic Study, you have been randomly assigned to receive genetic services by telephone.

You will speak with a Penn Medicine genetic counselor by telephone from a private office at the [NAME OF INSTITUTION]. Please contact the Penn TeleGenetics Clinic Study team to schedule your appointment:

[NAME OF RESEARCH TEAM, PHONE NUMBER AND EMAIL]


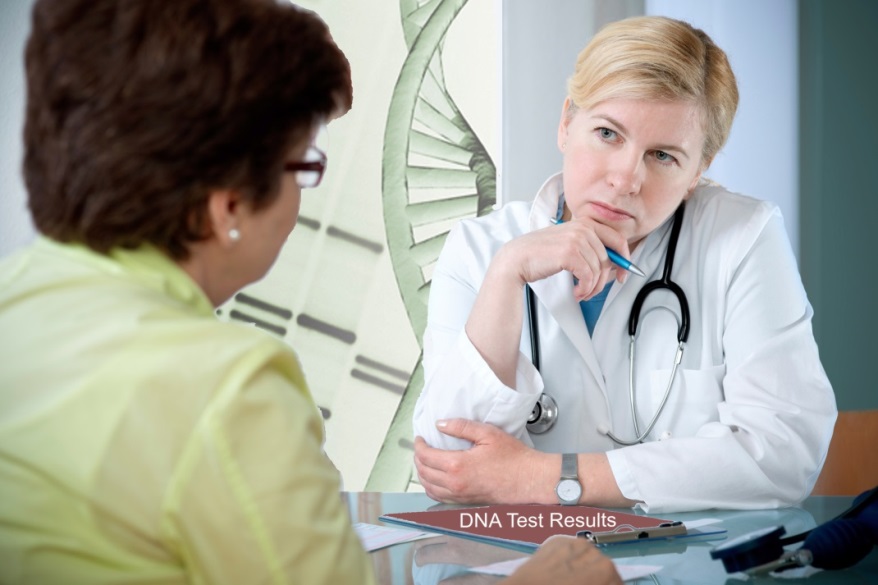


If you have any questions, contact:

[NAME OF SITE STAFF, PHONE NUMBER AND EMAIL]
