# Supplementary material for: Randomized study of remote telehealth genetic services versus usual care in oncology practices without genetic counselors
Source: Cancer Med. 2021 Jun 8;10(13):4532–41. doi: 10.1002/cam4.3968 (PMC8267134; doi:10.1002/cam4.3968)
Supplement: Supplementary file 2 — Table S1 [file CAM4-10-4532-s002.docx]

| Supplemental Table 1 Patient reported outcomes by phone v. videoconference remote services | | | | | | | | | | |
| --- | --- | --- | --- | --- | --- | --- | --- | --- | --- | --- |
|  | Phone (n=47) | | | | | Videoconference (n=49) | | | | |
|  | T0  Mean [SE] | T0-T1 Change [SE] | T0-T2  Change [SE] | T0-T3 Change [SE] | T0-T4 Change [SE] | T0  Mean [SE] | T0-T1  Change [SE] | T0-T2  Change [SE] | T0-T3  Change [SE] | T0-T4  Change [SE] |
| Knowledge (range 18-87) | 54.37 [1.95] | +5.13 [2.24] | +6.95 [2.23] | +6.7 [2.46] | +7.2 [2.16] | 45.27 [2.85] | +12.38 [2.83] | +15.94 [2.68] | +14.74 [3.12] | +13.07 [2.92] |
| Cancer- specific distress (range 0-70) | 22.75 [1.93] | -2.23 [1.97] | -1.05 [2.66] | -2.01 [2.87] | -0.68 [3.08] | 20.38 [2] | +0.46 [1.98] | -1.75 [2.26] | -1.59 [2.6] | +0.87 [2.66] |
| General Anxiety (range 0-21) | 7.74 [0.51] | -0.27 [0.52] | +0.04 [0.53] | +0.83 [0.78] | -0.15 [0.7] | 6.96 [0.5] | +0.15 [0.5] | -0.3 [0.46] | +0.05 [0.57] | -0.77 [0.56] |
| Depression (range 0-21) | 4.54 [0.5] | -0.19 [0.41] | -0.07 [0.48] | +0.46 [0.69] | -0.37 [0.61] | 3.78 [0.45] | +0.01 [0.41] | -0.31 [0.45] | +0.47 [0.64] | -0.3 [0.5] |
|  |  |  | T2  Mean [SE] | T2-T3  Change [SE] | T2-T4  Change [SE] |  |  | T2  Mean [SE] | T2-T3  Change [SE] | T2-T4  Change [SE] |
| Negative response* (range 0-30) |  |  | 4.97 [0.98] | +1.39 [1.52] | -0.26 [1.09] |  |  | 4.63 [1.01] | +1.63 [1.37] | -0.48 [1.02] |
| Positive response* (range 0-20) |  |  | 4.25 [0.77] | +1.4 [1] | +2.8 [1.16] |  |  | 3.93 [0.84] | +0.8 [0.86] | +1.13 [1.03] |
| Uncertainty* (range 0-45) |  |  | 7.3 [1.02] | +1.11 [1.29] | +1.02 [1.02] |  |  | 6.47 [0.98] | +2.06 [1.24] | +1.69 [1.1] |
|  |  | T1  mean [SE] | T2  mean [SE] |  |  |  | T1  mean [SE] | T2  mean [SE] |  |  |
| Satisfaction -Genetic Services* (range 12-60) |  | 48.45 [1.01] | 50.08 [1.35] |  |  |  | 48.54 [1.16] | 49.15 [1.24] |  |  |
| Satisfaction – Telemedicine* (range 10-50) |  | 43.95 [0.74] | 44.70 [0.82] |  |  |  | 42.67 [0.74] | 43.64 [0.68] |  |  |
| No significant differences in change scores between arms after adjusting for baseline literacy, knowledge, depression, history of cancer, education and income.  *There were no significant differences between arms at T1 or T2 | | | | | | | | | | |
