# Supplementary material for: Randomized study of remote telehealth genetic services versus usual care in oncology practices without genetic counselors
Source: Cancer Med. 2021 Jun 8;10(13):4532–41. doi: 10.1002/cam4.3968 (PMC8267134; doi:10.1002/cam4.3968)
Supplement: Supplementary file 3 — Table S2 [file CAM4-10-4532-s003.docx]

**Supplemental Table 2: Additional details of Patient reported outcomes, including percent with non-missing data. T0 means differ from Supplemental Table 1 due to the use of the second baseline among those rerandomized for the baseline reported in that table.**

| \|  \| Telephone \| Videoconference \| \| --- \| --- \| --- \| \|  \| (N = 47) \| (N = 49) \| |
| --- | --- | --- | --- | --- | --- | --- |
| \| **Knowledge T0** \|  \|  \| \| --- \| --- \| --- \| \| Mean (SD) \| 52.39 (15.11) \| 43.66 (20.11) \| \| N (% Non-missing) \| 47 (100.0%) \| 48 (98.0%) \| \| **Cancer-specific Distress T0** \|  \|  \| \| Mean (SD) \| 23.38 (15.40) \| 20.56 (14.68) \| \| N (% Non-missing) \| 47 (100.0%) \| 49 (100.0%) \| \| **General Anxiety T0** \|  \|  \| \| Mean (SD) \| 7.85 (3.66) \| 6.96 (3.71) \| \| N (% Non-missing) \| 47 (100.0%) \| 49 (100.0%) \| \| **Depression T0** \|  \|  \| \| Mean (SD) \| 4.39 (3.37) \| 3.92 (3.49) \| \| N (% Non-missing) \| 47 (100.0%) \| 49 (100.0%) \| \| **Knowledge T1** \|  \|  \| \| Mean (SD) \| 59.43 (14.20) \| 59.15 (10.74) \| \| N (% Non-missing) \| 35 (74.5%) \| 37 (75.5%) \| \| **Cancer-specific Distress T1** \|  \|  \| \| Mean (SD) \| 19.69 (14.64) \| 18.68 (13.37) \| \| N (% Non-missing) \| 34 (72.3%) \| 37 (75.5%) \| \| **General Anxiety T1** \|  \|  \| \| Mean (SD) \| 7.30 (3.89) \| 6.43 (3.56) \| \| N (% Non-missing) \| 35 (74.5%) \| 38 (77.6%) \| \| **Depression T1** \|  \|  \| \| Mean (SD) \| 4.46 (4.29) \| 3.34 (3.04) \| \| N (% Non-missing) \| 35 (74.5%) \| 38 (77.6%) \| \| **Knowledge T2** \|  \|  \| \| Mean (SD) \| 60.30 (9.55) \| 60.37 (10.16) \| \| N (% Non-missing) \| 27 (57.4%) \| 36 (73.5%) \| \| **Cancer-specific Distress T2** \|  \|  \| \| Mean (SD) \| 21.93 (16.68) \| 16.53 (14.06) \| \| N (% Non-missing) \| 27 (57.4%) \| 36 (73.5%) \| \| **General Anxiety T2** \|  \|  \| \| Mean (SD) \| 6.66 (3.88) \| 5.86 (4.04) \| \| N (% Non-missing) \| 27 (57.4%) \| 37 (75.5%) \| \| **Depression T2** \|  \|  \| \| Mean (SD) \| 4.67 (4.13) \| 2.50 (2.81) \| \| N (% Non-missing) \| 27 (57.4%) \| 38 (77.6%) \| \| **Negative Response T2** \|  \|  \| \| Mean (SD) \| 3.52 (5.18) \| 2.93 (5.56) \| \| N (% Non-missing) \| 27 (57.4%) \| 34 (69.4%) \| \| **Positive Response T2** \|  \|  \| \| Mean (SD) \| 3.37 (3.99) \| 2.46 (3.81) \| \| N (% Non-missing) \| 27 (57.4%) \| 35 (71.4%) \| \| **Uncertainty T2** \|  \|  \| \| Mean (SD) \| 6.43 (6.06) \| 5.76 (6.03) \| \| N (% Non-missing) \| 27 (57.4%) \| 35 (71.4%) \| \| **Knowledge T3** \|  \|  \| \| Mean (SD) \| 59.18 (12.76) \| 60.56 (10.01) \| \| N (% Non-missing) \| 25 (53.2%) \| 32 (65.3%) \| \| **Cancer-specific Distress T3** \|  \|  \| \| Mean (SD) \| 17.87 (13.98) \| 16.27 (13.34) \| \| N (% Non-missing) \| 25 (53.2%) \| 31 (63.3%) \| \| **General Anxiety T3** \|  \|  \| \| Mean (SD) \| 7.10 (4.36) \| 5.81 (3.59) \| \| N (% Non-missing) \| 25 (53.2%) \| 32 (65.3%) \| \| **Depression T3** \|  \|  \| \| Mean (SD) \| 4.15 (4.52) \| 3.10 (2.69) \| \| N (% Non-missing) \| 25 (53.2%) \| 32 (65.3%) \| \| **Negative Response T3** \|  \|  \| \| Mean (SD) \| 3.30 (6.88) \| 3.59 (5.15) \| \| N (% Non-missing) \| 25 (53.2%) \| 32 (65.3%) \| \| **Positive Response T3** \|  \|  \| \| Mean (SD) \| 3.88 (5.13) \| 2.28 (3.49) \| \| N (% Non-missing) \| 25 (53.2%) \| 32 (65.3%) \| \| **Uncertainty T3** \|  \|  \| \| Mean (SD) \| 6.96 (7.07) \| 7.00 (6.29) \| \| N (% Non-missing) \| 25 (53.2%) \| 32 (65.3%) \| \| **Knowledge T4** \|  \|  \| \| Mean (SD) \| 61.00 (10.17) \| 59.45 (9.32) \| \| N (% Non-missing) \| 24 (51.1%) \| 32 (65.3%) \| \| **Cancer-specific Distress T4** \|  \|  \| \| Mean (SD) \| 21.01 (14.49) \| 17.59 (13.86) \| \| N (% Non-missing) \| 23 (48.9%) \| 32 (65.3%) \| \| **General Anxiety T4** \|  \|  \| \| Mean (SD) \| 6.50 (3.76) \| 5.67 (3.32) \| \| N (% Non-missing) \| 24 (51.1%) \| 33 (67.3%) \| \| **Depression T4** \|  \|  \| \| Mean (SD) \| 3.96 (3.37) \| 2.88 (2.58) \| \| N (% Non-missing) \| 24 (51.1%) \| 33 (67.3%) \| \| **Negative Response T4** \|  \|  \| \| Mean (SD) \| 3.38 (5.62) \| 2.36 (3.18) \| \| N (% Non-missing) \| 24 (51.1%) \| 33 (67.3%) \| \| **Positive Response T4** \|  \|  \| \| Mean (SD) \| 6.08 (6.21) \| 3.67 (4.85) \| \| N (% Non-missing) \| 24 (51.1%) \| 33 (67.3%) \| \| **Uncertainty T4** \|  \|  \| \| Mean (SD) \| 6.31 (6.38) \| 6.96 (7.30) \| \| N (% Non-missing) \| 24 (51.1%) \| 33 (67.3%) \| \| **Knowledge Re-Randomized Second Baseline** \|  \|  \| \| Mean (SD) \| 47.70 (20.57) \| 46.69 (24.77) \| \| N (% Non-missing) \| 10 (21.3%) \| 11 (22.4%) \| \| **Cancer-specific Distress Re-Randomized Second Baseline** \|  \|  \| \| Mean (SD) \| 24.00 (11.31) \| 22.00 (14.89) \| \| N (% Non-missing) \| 10 (21.3%) \| 11 (22.4%) \| \| **General Anxiety Re-Randomized Second Baseline** \|  \|  \| \| Mean (SD) \| 8.10 (4.20) \| 7.74 (3.23) \| \| N (% Non-missing) \| 10 (21.3%) \| 11 (22.4%) \| \| **Depression Re-Randomized Second Baseline** \|  \|  \| \| Mean (SD) \| 6.70 (4.37) \| 4.92 (3.79) \| \| N (% Non-missing) \| 10 (21.3%) \| 11 (22.4%) \| \| **Satisfaction-Genetic Services T1** \|  \|  \| \| Mean (SD) \| 49.13 (5.98) \| 49.27 (6.37) \| \| N (% Non-missing) \| 35 (74.5%) \| 37 (75.5%) \| \| **Satisfaction-Genetic Services T2** \|  \|  \| \| Mean (SD) \| 52.38 (5.61) \| 50.15 (6.49) \| \| N (% Non-missing) \| 27 (57.4%) \| 37 (75.5%) \| \| **Satisfaction–Telemedicine T1** \|  \|  \| \| Mean (SD) \| 44.07 (4.41) \| 42.98 (4.11) \| \| N (% Non-missing) \| 35 (74.5%) \| 37 (75.5%) \| \| **Satisfaction–Telemedicine T2** \|  \|  \| \| Mean (SD) \| 46.11 (3.25) \| 43.59 (4.03) \| \| N (% Non-missing) \| 27 (57.4%) \| 37 (75.5%) \| |
